# The effects of irradiation on the biological and biomechanical properties of an acellular porcine superflexor tendon graft for cruciate ligament repair

**DOI:** 10.1002/jbm.b.33786

**Published:** 2016-09-23

**Authors:** Jennifer H. Edwards, Anthony Herbert, Gemma L. Jones, Iain W. Manfield, John Fisher, Eileen Ingham

**Affiliations:** ^1^ Institute of Medical and Biological Engineering University of Leeds Leeds UK; ^2^ School of Molecular and Cellular Biology University of Leeds Leeds UK

**Keywords:** anterior cruciate ligament, knee reconstruction, decellularization, sterilization, acellular graft

## Abstract

Acellular xenogeneic tissues have the potential to provide ‘off‐the‐shelf’ grafts for anterior cruciate ligament (ACL) repair. To ensure that such grafts are sterile following packaging, it is desirable to use terminal sterilization methods. Here, the effects of gamma and electron beam irradiation on the biological and biomechanical properties of a previously developed acellular porcine superflexor tendon (pSFT) were investigated. Irradiation following treatment with peracetic acid was compared to peracetic acid treatment alone and the stability of grafts following long‐term storage assessed. Irradiation did not affect total collagen content or biocompatibility (determined using a contact cytotoxicity assay) of the grafts, but slightly increased the amount of denatured collagen in and decreased the thermal denaturation temperature of the tissue in a dose dependant fashion. Biomechanical properties of the grafts were altered by irradiation (reduced ultimate tensile strength and Young's modulus, increased failure strain), but remained superior to reported properties of the native human ACL. Long term storage at 4°C had no negative effects on the grafts. Of all the conditions tested, a dose of minimum 25 kGy of gamma irradiation had least effect on the grafts, suggesting that this dose produces a biocompatible pSFT graft with adequate mechanical properties for ACL repair. © 2016 Wiley Periodicals, Inc. J Biomed Mater Res Part B: Appl Biomater, 105B: 2477–2486, 2017.

## INTRODUCTION

Damage to the anterior cruciate ligament (ACL) is increasingly common amongst a young and active population. For a severe ACL tear, there is a need to replace the ligament to restore stability within the joint. There are at least 100,000 ACL reconstructions performed per year in North America^1^ and over 7000 per year in the UK.^2^ Gold standard treatment for ACL reconstruction is the use of autograft or allograft tissues.^3^ The surgical use of autograft has limitations including donor site morbidity,^4^ leading to the increasing use of allografts.^5^ Allografts are however associated with prolonged periods of incorporation due to the presence of cellular components.^6^ A promising alternative is the use of acellular allogeneic or xenogeneic tendon or ligament tissue for ACL reconstruction. Removal of immunogenic cells from such tissue has the potential to create a biocompatible decellularized biological scaffold which, once implanted, will not be subject to initial tissue degradation due to adverse immunological responses or graft necrosis and which will regenerate with the recipients endogenous cells over time.^7^, ^8^

Decellularized biological scaffolds derived from human donor and animal tissues are used extensively in surgical applications.^9^, ^10^ These are largely produced from porcine small intestinal submucosa, human and bovine dermis, or pericardium.^11^ There is increasing evidence of the advantages of tissue specific decellularized biological scaffolds, such as providing appropriate biomechanical properties or inducing appropriate cellular differentiation and constructive remodelling.^11^ We have developed processes for the production of tissue specific biological scaffolds which preserve the physical properties, thus delivering multiscale hierarchical matrix architectures that replicate tissue‐specific biomechanical and biological functions. The process uses low concentration sodium dodecyl sulphate (SDS) and proteinase inhibitors and has been successfully applied to cardiovascular tissues,^8^, ^12^ musculoskeletal tissues,^13^ and dermis,^14^ with evidence of the mechanisms of action^8^, ^15^ and clinical utility of this approach now emerging.^16^, ^17^ The process has been adopted to the development of a decellularized porcine superflexor tendon (pSFT).^18^ The resultant biological scaffold has been shown to possess the mechanical properties necessary for deployment as an “off the shelf” alternative to current methods of ACL reconstruction.

Currently, the decellularized pSFT is chemically sterilized using peracetic acid (PAA; 0.1% v/v), a process used previously for the sterilization of biological grafts^19^, ^20^ and shown to effectively kill resistant bacterial spores in a decellularized biological scaffold.^21^ However, chemical sterilization requires further processing into packaging for storage, distribution and use, creating a risk of contamination poststerilization. To ensure product safety, terminal sterilization of the packaged product is highly desirable.

For donor human tissue, grafts are often sterilized by gamma irradiation at a minimum 25 kGy dosage, but this has been shown to adversely affect tissue biomechanics.^22^, ^23^, ^24^ For products with a very low bioburden a lower dose of 15 kGy (VDMax 15) is acceptable (ISO 11137‐2‐212), as would be the case for grafts which have undergone PAA treatment. More recently, the use of electron beam (E‐beam) irradiation sterilization has increased,^25^, ^26^ and has been shown to have different effects on the tissue biomechanics compared to gamma irradiation.^27^ These differences are thought to be a function of the higher dose rate of E‐beam compared to gamma irradiation, reducing the sterilization time and detrimental effects on the tissue. It is also important to consider irradiation following specific decellularization protocols, as different decellularization methods may alter tissue components and may affect further changes during irradiation.

The purpose of this study was to compare the biological and biomechanical properties of decellularized pSFT (d‐pSFT) that had been treated with PAA to those treated with PAA before irradiation with one of several irradiation processes, including high (55 and 30 kGy gamma; 34 kGy E‐beam) and low dose gamma and E‐beam (15 kGy) and fractioned E‐beam (15 + 15 kGy). Structural and chemical analysis of the tissues was used to investigate collagen structure following the irradiation treatments. Biocompatibility of the tissues was assessed through cytotoxicity assays since ionising irradiation can create toxic by‐products.^24^ Uniaxial tensile testing to failure was applied to understand the effects of the irradiation treatments on the biomechanical properties of the tissues. To assess time‐dependant degradation following treatment, tissues were analyzed within 1 month of irradiation and following storage at 4°C for 12 months.

## METHODS

### Materials

Unless otherwise stated, all reagents used during the study were obtained from Sigma‐Aldrich (Dorset, UK), VWR International (Lutterworth, UK) or Fisher Scientific (Leicestershire, UK). Porcine superflexor tendons (pSFT) were harvested from the hind limbs of 4 month old Large White pigs obtained from a local abattoir (J. Penny, Leeds, UK) within 24 h of slaughter.

### Preparation and decellularization of porcine superflexor tendons (pSFT)

The superflexor tendon was located (running from toe to ankle) following removal of the skin and subcutaneous tissue. The tendon was cleaned of connective tissue *in situ* and dissected from the foot. One branch of the isolated pSFT was removed before storage at −80°C on filter article moistened with phosphate buffered saline (PBS; Oxoid).pSFT were decellularized in batches of 12–24 following a previously established method.^18^ Briefly, the frozen pSFTs were thawed and then subjected to two freeze thaw cycles (−80°C) in hypotonic buffer (10 m*M* tris, 2.7 m*M* EDTA, 10 KIU mL^−1^ aprotinin [Mayfair House, Leeds, UK], pH 8.0). Tendons were then washed in acetone (50 mL) three times for 1 h at 42°C with agitation (120 rpm, PSU‐10i Orbital shaking platform, Grant Instruments, UK) to remove fat before rehydration in five changes of PBS (50 mL). Subsequent washes were carried out using 100 mL of solution per tendon in roller bottles (CELLROLL apparatus, INTEGRA Biosciences AG, UK; 12 tendons per bottle, 6 rpm) at 37°C. The pSFT were washed in hypotonic buffer for 24 h followed by hypotonic buffer plus 0.1% (w/v) SDS. These two washes were repeated. Four washes in PBS (2× 30 min, 70 h and 30 min) were carried out prior to incubation in nuclease solution (50 m*M* tris buffer, 1 m*M* MgCl_2_·6H_2_O, 1 U mL^−1^ Benzonase (Merck KGaA, Germany, pH 7.6; 60 mL per tendon) for 2 h. This step was repeated twice more before three washes in PBS containing EDTA (2.7 m*M* EDTA) and an overnight wash in hypertonic buffer (50 m*M* tris, 1.5*M* sodium chloride). Three 30 min PBS washes were carried out prior to sterilization with peracetic acid (0.1%; v/v; pH 6.0) for 3 h. Final PBS washes (3 × 30 min, 2 × 60 h, and 1 × 120 h) were carried out prior to packaging.

All tendons were packaged aseptically in specially designed foil/Tyvek pouches (Riverside Medical Packaging Company, Derby, UK). Packaging was suitable for gamma and E‐beam irradiation and allowed the tendons to be stored flat at −80°C prior to irradiation.

**Table 1:** Observations from Histological Sections Stained with H&E and Sirius Red

	0 months	12 months
PAA	Clear neat crimp, no bundle separation.	Consistent bundles with sharp crimp. Some holes in one section.
30 kGy gamma	Clear crimp. Neat bundles, slight separation in some samples.	Clear crimp, neat sections, holes in one sample. Some smoothing.
55 kGy gamma	Clear crimp, smoothing in many samples. Some bundle separation and disruption.	Clear crimp on all samples. Some smoothing, bundle separation in most samples. Holes visible in one sample
34 kGy E‐beam	Clear crimp, some smoothed regions and slight separation of bundles in some sections. Some small holes noted.	Clear crimp, neat bundles. Some separation and smoothing present in most sections.
15 kGy gamma	Clear, neat bundles with sharp crimp.	Neat bundles, clear crimp, slight smoothing.
15 kGy E‐beam	Clear crimp, some smoothing, and bundle separation, patches of holes in two samples.	Clear crimp, neat bundles, some separation, and smooth regions.
(15 + 15) kGy E‐beam	Good crimp visible in most sections with some smoothing and bundle separation. One section showed prolific holes.	Neat bundles, good crimp. Slight bundle separation and some smoothing. Holes in three samples, prolific in one case.

Comments have been collated from images across three tendon regions and at least three tendons per group.

### Irradiation sterilization of decellularized pSFTs

All decellularized pSFT (d‐pSFT) included in the study were sterilized using PAA prior to packaging and storage at −80°C as described above. For irradiation, d‐pSFT were removed from frozen storage and shipped under refrigerated conditions to Synergy Health PLC (Swindon, UK). Seventy‐two d‐pSFT were irradiated in replicates of *n* = 12 using 30 kGy gamma (30G), 55 kGy gamma (55G), 34 kGy E‐beam (34E), 15 kGy gamma (15G), 15 kGy E‐beam (15E), or (15 + 15) kGy E‐beam (fractionated dose, 15 + 15E). Following irradiation, samples were stored at 4°C and analyzed immediately postirradiation (0 months; *n* = 6) and after 12 months (*n* = 6) storage at 4°C. Irradiation doses had a tolerance of ±10%. Control, nonirradiated d‐pSFT (PAA) were placed in storage at 4°C when irradiated tendons were shipped (to match the refrigerated storage time of irradiated tendons) and analyzed at the same time points as above.

### Histological analysis

Samples of the ankle, middle and toe regions of each d‐pSFT were fixed in 10% (v/v) neutral buffered formalin (NBF) for 96 h. Fixed samples were processed automatically (Leica 11020 Tissue processor) and embedded in paraffin wax using standard techniques. Two samples of each tendon region were embedded to provide longitudinal and transverse tissue sections. Sections were cut at 10 µm to prevent disruption of the collagen crimp and stained with haematoxylin and eosin (H&E) or Sirius red in saturated picric acid. Sections were viewed under bright field (H&E) or polarized light (Sirius red) microscopy and images were captured using a Zeiss Axio Imager 2 microscope (Zeiss, UK).

### Quantification of total collagen content and denatured collagen content

The hydroxyproline (HYP) content of acid hydrolyzed tissue samples was used as a measure of the total collagen content of d‐pSFT tissue. Tissue from the ankle, middle and toe regions of the tendons was lyophilized to constant weight using a Thermo Savant ModulyoD freeze drier. Approximately 30 mg samples of freeze‐dried tissue were hydrolysed by autoclaving (121°C for 4 h) in 5 mL of 6*M* hydrochloric acid (HCl) before neutralization with 6*M* sodium hydroxide. HYP content of the digests was measured in triplicate using the method described by Edwards & O'Brien.^28^ Briefly, 50 µL of test solution was placed in a flat‐bottomed 96‐well plate. Oxidizing solution (50 µL; 705 mg chloramine T, 50 mL distilled water) was added to each well and shaken gently for 5 min. Ehrlich's reagent (100 µL;7.5 g *p*‐dimethylaminobenzaldehyde, 30 mL propan‐1‐ol and 13 mL 62% (v/v) perchloric acid) was added to each well and mixed thoroughly. The plate was sealed and incubated at 60°C for 45 min. Absorbance was read at 570 nm and HYP content calculated as µg HYP per mg dry weight tissue. A standard curve of trans‐4‐hydroxy‐L‐proline was included in each plate.

Denatured collagen content of the d‐pSFT tissue was assessed by treatment with α‐chymotrypsin. The alpha‐chymotrypsin enzyme used in the denatured collagen assay preferentially cleaves amine bonds around hydrophobic amino acids.^29^ Digestion of tissue with the enzyme releases small fragments of peptide chain into solution, which can be quantified using the hydroxyproline assay. An increase in the amount of hydroxyproline in this digest indicates disruption of the bonding within the peptide chain, allowing the enzyme to release larger or more numerous fragments into solution. Approximately 60 mg of finely macerated, freeze dried tissue was incubated in 5 mL of α‐chymotrypsin solution (bovine pancreas, 40 U mg^−1^ in 0.1*M* Tris plus 1.5 mg mL^−1^ CaCl_2_; pH 7.8) for 24 h at 37°C. Following digestion, the samples were centrifuged and 4 mL of the resulting supernatant removed. Supernatant was hydrolysed using 4 ml 6M HCL before neutralization and measurement of HYP content as above.

### Measurement of thermal stability

Thermal stability of the d‐pSFT tissue was assessed using differential scanning calorimetry (DSC) and static light scattering (SLS). For DSC analysis, approximately 5 mg samples of tissue (ankle region) were analyzed on a Q2000 DSC Instrument (TA Instruments). Samples were heated from 15 to 130°C at a rate of 4°C per minute in hermetically sealed aluminium pans. Transition temperature was the temperature at which the heat flow through the sample was highest. SLS analysis was carried out using an Optim 1000 (Avacta Analytical), with a small longitudinal sample of tendon in the microcuvette. Samples were heated from 10 to 90°C in 1°C increments and the SLS at 266 nm recorded for each temperature. Transition temperature was taken as the midpoint of the sudden decline in SLS.

### *In vitro* biocompatibility tests

The biocompatibility of d‐pSFT tissue was investigated using the *in vitro* contact cytotoxicity assay with L929 (Health Protection Agency) and BHK (BHK 21 strain 31, Health Protection Agency) cell lines. L929 cells were maintained in Dulbecco's modified Eagle's medium (DMEM) supplemented with 10% (v/v) fetal calf serum (FBS; Sera Lab), 100 U mL^−1^ penicillin, 100 µg mL^−1^ streptomycin and 2 m*M* l‐glutamine. BHK culture medium consisted of Glasgow's minimal essential media (GMEM) supplemented with 5% (v/v) FBS, 10% (v/v) tryptose phosphate broth, 100 U mL^−1^ penicillin, 100 µg mL^−1^ streptomycin, and 2 m*M* l‐glutamine. Cells were cultured at 37°C in a humidified atmosphere of 5% (v/v) CO_2_ in air.

Sections of d‐pSFT tissue (approx. 2 × 2 × 5 mm) from the mid region of each tendon were attached to the centre of one well in 6 well tissue culture plates and secured in place using Steri‐Strip SkinClosure (Medisave). Duplicate plates allowed each d‐pSFT to be cultured with both L929 and BHK cells. Cell only, positive (cyanoacrylate contact adhesive) and negative (Steri‐Strip only) controls were included for each cell type. Plates were washed 3 times with PBS prior to seeding at a density of 5 × 10^5^ cells per well in their respective medium and cultured in a humidified atmosphere of 5% (v/v) CO_2_ in air. After 48 h, all wells were examined using phase contrast microscopy. The presence of cells growing up to the tissue was recorded, as well as any unusual features or changes in cell morphology. Wells were washed once with PBS before fixation in 10% (v/v) NBF for 10 min. NBF was removed and the cells stained with Giemsa for 5 min. All wells were washed with tap water until clear prior to observation and imaging under brightfield microscopy.

### Uniaxial tensile testing

For biomechanical testing, a portion of the tendon between ankle and middle regions was isolated and stored at 4°C prior to further shaping on the same day. Mechanical testing was carried out following the method described previously by Herbert et al.^18^ In brief, the tendons were placed on dry ice and processed into a dumbbell shape (working cross‐sectional area 3.5 × 5 mm, gauge length 30 mm). Prior to testing, specimens were wrapped in PBS soaked filter article and allowed to equilibrate to room temperature. Testing was carried out on an Instron 3365 (Instron, Bucks, UK) materials testing machine equipped with a 1000 N load cell. Bespoke cryo grips were used to attach the specimens to the testing machine and prevent slippage of the graft. A preload of 0.5 N was applied, followed by 10× preconditioning cycles between 0 and 5% strain at a displacement rate of 15 mm min^−1^ and finally a ramp to failure at a rate of 30 mm min^−1^. Failure was defined as midsubstance rupture. Load and extension data were acquired at a rate of 10 Hz and converted to stress–strain. The stress–strain behaviour of the specimens was analyzed by means of the Young's modulus (*E*), ultimate tensile strength (*σ*_UTS_), and failure strain (*ε*_UTS_).

### Data analysis

Statistical analysis was performed using GraphPad Prism software (GraphPad Software, CA, USA). Ordinary two‐way ANOVA was used to determine statistical variations between irradiation groups at both time points (0 & 12 months). In each case, Tukey's significant difference test was used for *post hoc* analysis, with a *p* values of <0.05 considered statistically significant. A Pearson correlation test (two‐tailed test, *p *<* *0.05) was used to check for correlation between the DSC and SLS transition temperatures.

## RESULTS

### Histological observations

**Figure 1:**
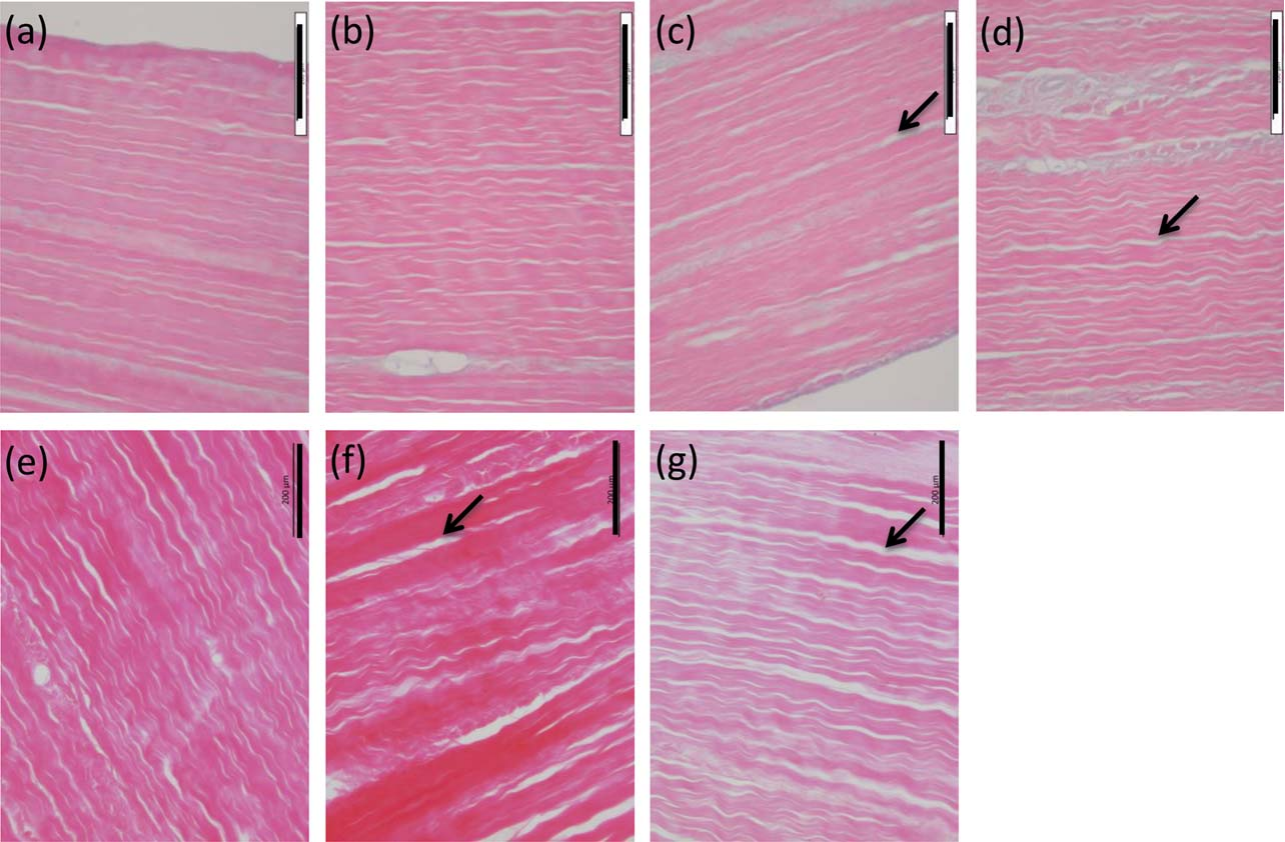
Histological structure of pSFT at 0 months post‐irradiation following Haematoxylin and Eosin staining. Longitudinal sections. Images show PAA only (a), 30G (b), 55G (c), 34E (d), 15G (e), 15E (f), and 15 + 15E (g). Arrows indicate regions of increased bundle separation, scale bars are 200 µm and images are representative of each condition at 0 and 12** **months.

**Figure 2:**
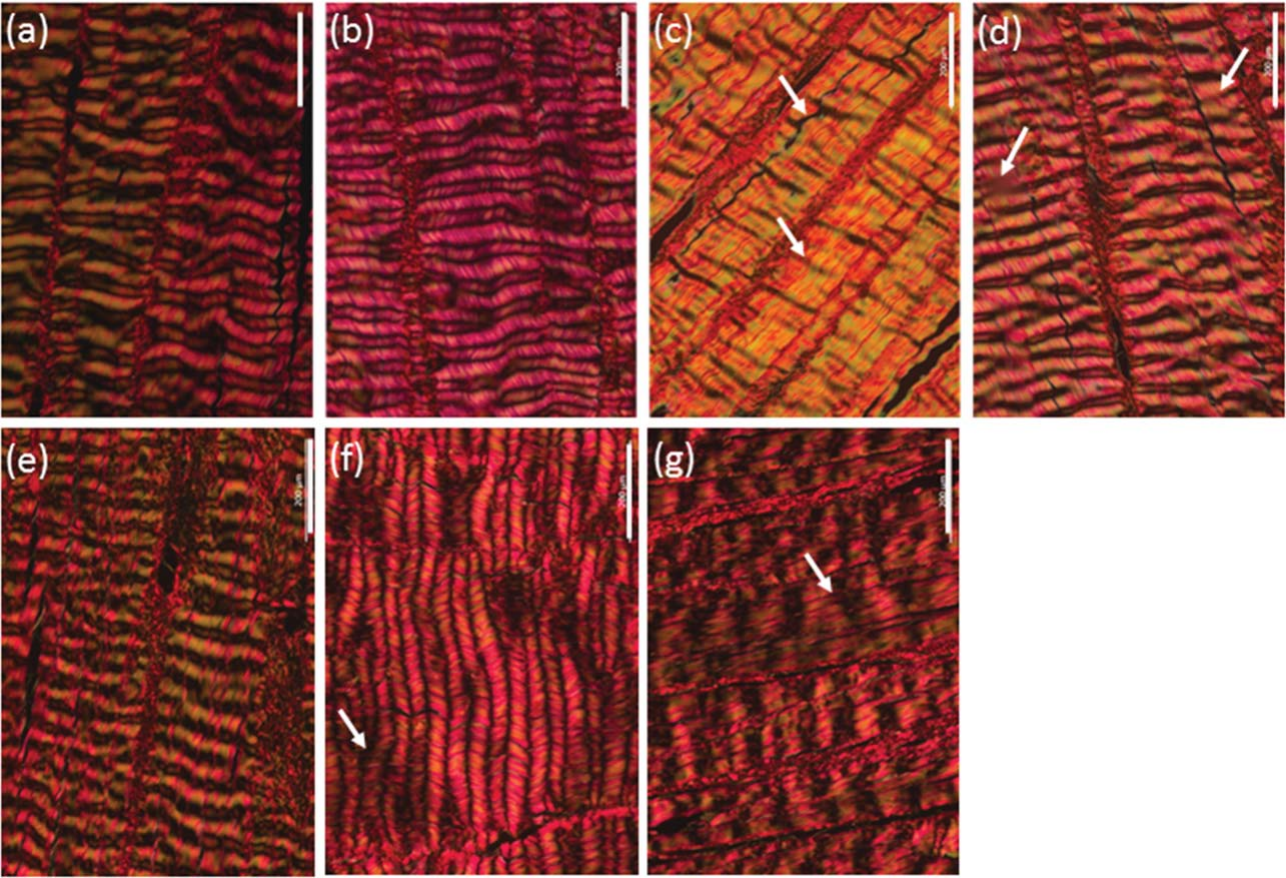
Histological structure of pSFT at 0 months post‐irradiation following Sirius red staining. Longitudinal sections imaged using polarized light microscopy. Images show PAA only (a), 30G (b), 55G (c), 34E (d), 15G (e), 15E (f), and 15 + 15E (g). Arrows indicate regions of flattened crimp, scale bars are 200 µm and images are representative of each condition at 0 and 12** **months.

Histological evaluation of d‐pSFT tissues at the 0 month time point revealed several differences in the arrangement of collagen fibres between tendon groups. Observations are summarised in Table 1 and images of the longitudinal sections after 0 months storage are presented in Figures 1 and 2. All histological sections stained with Sirius red and viewed under polarized light microscopy showed regions of tight, sharp crimp in longitudinal sections and fibre bundles were clearly visible in transverse sections. In tissues from the PAA group there were very few regions where the crimp was flattened. All irradiated d‐pSFTs showed more regions of fiber smoothing than PAA alone. This was most severe in tendons subjected to 55G [Figure 2(c)] and 15 + 15E irradiation [Figure 2(k)]. In some samples, the presence of holes within the tissue was noted [34E, 15E, 15 + 15E (0M), and 30G, 55G, 15 + 15E and PAA (12M)]. This was usually limited to one region and sample per group, but for the 15 + 15E group at 12 months post‐irradiation was observed in half of the samples. There were no observable changes in the tissue structure of any of the irradiated d‐pSFT following storage for 12 months.

### Total and denatured collagen content

**Figure 3:**
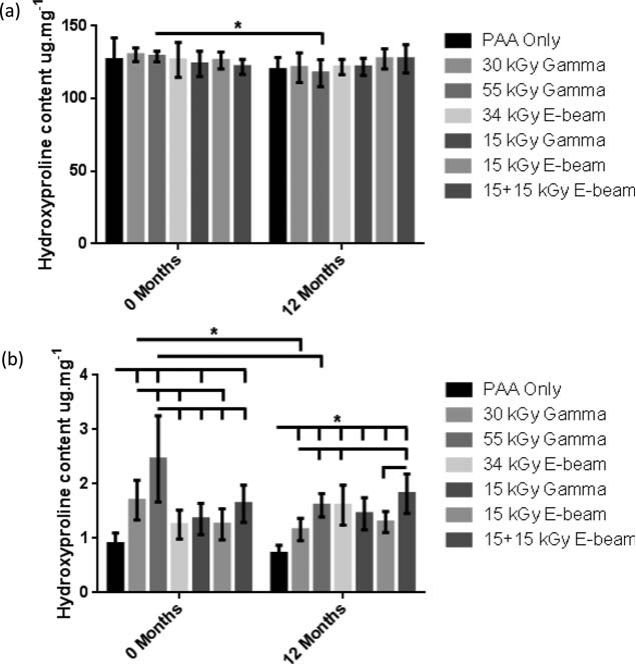
Hydroxyproline content of pSFT (a) and of α‐chymotrypsin digest of pSFT (denatured collagen content, (b)) following irradiation sterilization 0 and 12 months poststorage. A dose‐dependent increase in the amount of denatured collagen compared to the PAA only group was observed at 0 months post‐irradiation. At 12 months, the denatured collagen content of 30 and 55 kGy gamma irradiated samples was significantly lower than the same condition at 0 months. Data are presented as the mean (*n* = 6) ± 95% CI. Data was analyzed by 2‐way ANOVA, * denotes significant difference (*p* < 0.05) between linked groups.

Total collagen content and denatured collagen content of the ankle, middle, and toe regions of the d‐pSFT subject to each irradiation process was measured for all tendon groups. The HYP content (representative of total collagen) was found to be consistent across the ankle, middle, and toe regions for all conditions and storage times. There were some minor regional differences between ankle and middle regions at 0 and 12 months but these were not associated with a particular irradiation process and were considered negligible. Hence, in order to assess the overall effects of the different irradiation methods on the total and denatured collagen content of the d‐pSFTs, data for the different regions of each tendon was pooled for analysis. The effects of the irradiation processes on HYP and denatured collagen content of the d‐pSFT tissue at 0 and 12 months postirradiation is shown in Figure 3. HYP content data was analyzed by 2‐way analysis of variance, revealing no significant variation in HYP content for the different irradiation processes at 0 or 12 months storage at 4°C. There was a small but significant (*p* < 0.05) reduction in HYP content of the tissue treated with 55G between 0 and 12 months [Figure 3(a)] but no other significant effects of storage on the HYP content of the tissues.

The levels of HYP in the supernatant following the treatment of the tissue with α‐chymotrypsin (representative of denatured collagen) were extremely low (<3 µg mg^−1^) in all tissue samples. Overall, irradiation resulted in small increased levels of denatured collagen in the d‐pSFT [Figure 3b]. At 0 months, the levels of denatured collagen in the PAA group were significantly (*p* < 0.05; 2‐way ANOVA) lower than those treated with 15G, 30G, 55G, and 15 + 15E irradiation. Treatment with 55G resulted in the greatest increase in denatured collagen in the tissue at 0 months. After 12 months storage at 4°C the denatured collagen levels in all of the irradiated d‐pSFTs were significantly (*p* < 0.05; 2‐way ANOVA) greater than the PAA group. Interestingly, after 12 months storage, the levels of denatured collagen in the d‐pSFT treated with 30G and 55G were significantly (*p* < 0.05; 2‐way ANOVA) reduced compared to the same conditions at 0 months. The data also revealed that reducing the dose of either gamma or E‐beam sterilization from 30 to 15 kGy did not reduce the denatured collagen content of the d‐pSFTs at 0 or 12 months.

### Thermal stability

**Figure 4:**
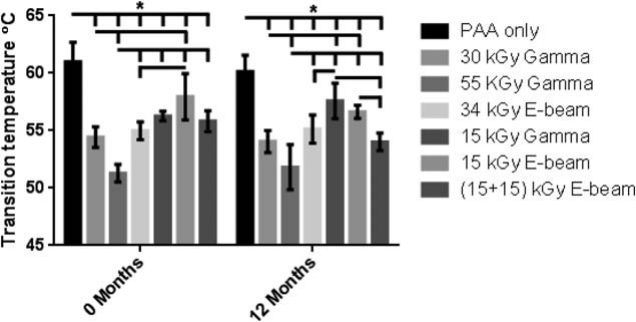
Thermal stability by DSF of decellularized pSFT samples at 0 and 12 months postirradiation. There was a significant difference in transition temperature between PAA only and all irradiation groups at 0 and 12** **months. There was an irradiation dose**‐**dependent decrease in the thermal transition temperature at both time points. Data is presented as the mean (*n* = 6)** **± 95% CI. Data was analyzed by 2‐way ANOVA * denotes significant difference (*p* < 0.05) between linked groups.

Two methods (DSC and SLS) for measuring the thermal stability of the d‐pSFT were initially assessed using samples from the PAA, 30G, 55G, and 34E groups at 0 months postirradiation. PAA treated d‐pSFTs had a transition temperature of 66.2 ± 2.0°C as measured by DSC and irradiation treatment resulted in a decrease in the transition temperature to 58.3 ± 1.0°C (30G), 54.9 ± 0.7°C (55G), and 58.2 ± 0.5°C (34E). Transition temperatures recorded using SLS were approx. 4°C lower in all tendon groups [5.2 ± 3.1°C (PAA), 3.8 ± 0.7°C (30G), 3.6 ± 0.7°C (55G), and 3.2 ± 1.0°C (34E)] and showed the same trend as those recorded by DSC. The two techniques were compared and showed excellent correlation (*R* ^2 ^= 0.9905, Pearson correlation test). For practical reasons, measurement of thermal properties for the d‐pSFT irradiated using 15G, 15E, and 15 + 15E and all tendons after 12 months storage was carried out by SLS. Data was analyzed by two‐way analysis of variance and is presented in Figure 4. Compared to PAA, all irradiated d‐pSFTs had significantly (*p* < 0.05) lower thermal transition temperatures at both the 0 and 12 month time points, with a dose‐dependent decrease in transition temperature with increasing dose of irradiation. Thermal transition temperature for the d‐SFT treated with 55G was significantly (*p* < 0.05) lower than all other groups at 0 months and significantly lower than the PAA, 30G, 15G, 34E, and 15E groups at 12 months post‐irradiation. No significant changes in the thermal temperature occurred during 12 months storage at 4°C.

### *In vitro* biocompatibility

**Figure 5:**
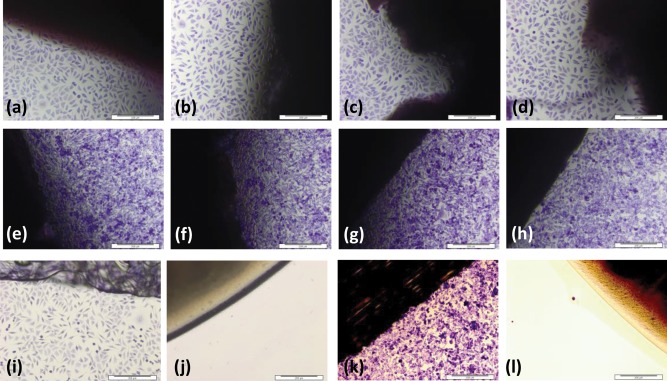
Giemsa stained cell cultures from contact cytotoxicity assays with 0 month post‐irradiation pSFT samples. Images show tissue samples with L929 (a–d) and BHK cells (e–h), as well as negative (steristrip only; i, k) and positive (cyanoacrylate; j, l) controls with the same cell types. PAA only (a, e), 30 kGy gamma (b, f), 55** **kGy gamma (c, g) and 34** **kGy E‐beam (d, h) groups are shown and images are representative of all other conditions and time points tested. Scale bars are 200 µm.

No toxicity of any of the d‐pSFT tissues, with or without irradiation, was observed. L929 and BHK cells grew up to and in contact with all tissue samples with no differences in cellular morphology compared to the cell only or Steri strip negative controls. For the positive controls, there was a void around the cyanoacrylate disc, with no attached cells visible. Representative images of the contact cytotoxicity tests carried out using tissues from the PAA, 30G, 55G, and 34E tendon groups are shown in Figure 5 at 0 months. These images are representative of the data for d‐pSFTs in all irradiation groups and storage times.

### Biomechanical testing

**Figure 6:**
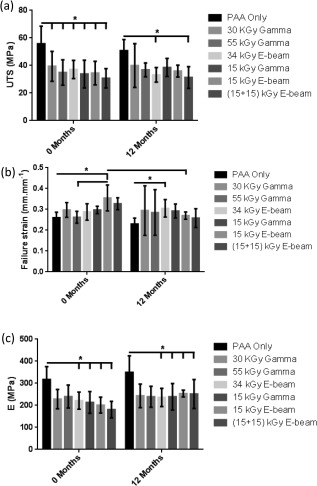
Biomechanical properties of the pSFT grafts following 0 and 12 months storage post‐irradiation. Data is presented as the mean ± 95% CI, *n* = 6 (except 30G and 55G at 12** **months, *n* = 3 and PAA 12** **months, *n* = 5). Data was analyzed by 2‐way ANOVA. * denotes significant difference (*p* < 0.05) between linked groups.

The uniaxial tensile testing results for all groups are presented in Figure 6. All of the irradiation treatments significantly (*p* < 0.05) reduced the UTS of the d‐pSFTs at 0 months compared to PAA. There were no significant differences between the UTS of the tissues treated by the different irradiation processes at 0 months. After 12 months storage, although the UTS recorded for the irradiated tissues was lower than the PAA tissue, this was only significant for the tissues that had been irradiated with 34E and 15 + 15E. There were no significant changes in the UTS in any group over 12 month storage at 4°C [Figure 5(a)].

There was a trend of slightly increased failure strain in the irradiated tendons compared to the PAA, however this was only significant (*p* < 0.05) at 0 months for tendons irradiated with 15E (vs. PAA and 55G) and at 12 months for those treated with 34E. Interestingly, the failure strain of the tendons treated with 15E was significantly lower at 12 month compared to 0 months storage at 4°C [Figure 5(b)].

The Young's modulus of the irradiated tendons was lower than that of the PAA tendons at both 0 and 12 months, however this only reached the *p *< 0.05 level of significance for the tendons subject to 34E, 15G, 15E, and 15 + 15E. There was no change in Young's modulus for any group following the 12 month period of storage at 4°C [Figure 5(c)].

## DISCUSSION

This study has shown that treatment of decellularized pSFT using gamma or E‐beam irradiation had several effects on the tissue properties. While irradiation had no effect on the capacity of two cell lines to grow up to and in contact with the tissue samples, histological analysis of tissue sections showed changes in the collagen structure. Effects included smoothing of some regions of the collagen crimp, slight separation of the bundles and, in some cases, holes within the tissue. All irradiation treated tendons showed some histological changes when compared to tendons treated with PAA alone. Smoothing of the collagen crimp and separation of bundles appeared to increase with irradiation dose for both gamma and E‐beam and was most apparent in d‐pSFT treated with 55G irradiation. Histological observations were supported by small, irradiation dose‐dependent increases in denatured collagen content of the tissues and reductions in the thermal transition temperatures. In addition, there were changes to the gross material properties of the d‐pSFT as a result of irradiation treatment, manifest as a reduction in UTS and Young's modulus compared to PAA, but these changes were not irradiation dose dependent. Long term storage at 4°C resulted in a slight reduction in denatured collagen content (30G, 55G) and failure strain (15E), but was not detrimental for any condition.

During sterilization with ionising radiation in the presence of water, homolytic fission produces a hydroxyl radical and a hydrogen atom (HO^•^ and H^•^, respectively.^30^) Radicals have an unpaired electron, making them highly reactive and microbicidal.^31^ Although the HO^•^ radical has a very short half‐life, it is highly reactive with a variety of biological molecules; attacking peptide bonds, propagating reactive oxygen species and causing further damage to proteins. In the case of E‐beam irradiation, the incident electrons can also interact with tissues as radicals.

These radicals, while necessary for microbial inactivation, may have detrimental effects on the collagen hierarchy in tendon tissue. Collagen is formed from the arrangement of tropocollagen monomers, themselves a triple helix arrangement of α‐chains. Hydroxylation of proline and lysine residues within the helix, as well as between tropocollagen units (as they assemble into collagen fibrils), provide stability to the structure.^32^ Fibrils associate into fibres via covalent bonding, and it is these fibres which display the crimp pattern characteristic of tendon tissue. Many studies have investigated the effects of irradiation on tendon tissue, providing insights into the mechanisms of degradation by free radicals. It has been reported that low‐dose gamma irradiation causes cleavage of the α‐chain (chain scission) and disruption of hydrogen bonding.^33^, ^34^ Covalent crosslinks, such as those between fibrils, have been shown to be more resistant to irradiation damage and crosslinks may remain following doses of 60 kGy gamma. In addition, when water is present within tissue during irradiation, intermolecular crosslinks may form, reducing the effects of chain scission compared to the effects of irradiation on freeze‐dried tissue.^34^, ^35^

The irradiation treatments applied during the present study resulted in small increases in the amount of denatured collagen present within the d‐pSFT. The magnitude of these changes was much smaller than the changes observed in transition temperature of the tissues. Transition temperature of tissues treated with 55 kGy gamma reduced by almost 10°C at 0 months, whereas the denatured collagen content was only increased by 1.5 µg mg^−1^. The α‐chymotrypsin enzyme used in the denatured collagen assay preferentially cleaves amine bonds around hydrophobic amino acids,^29^ which account for a low proportion of those found in porcine collagen.^36^ Although irradiation treatment would cause chain scission within tropocollagen molecules, bonding between fibres and fibrils may have prevented the α‐chymotrypsin from releasing long or numerous peptide fragments. Thermal transition occurs due to the breakdown of a protein's tertiary structure as it is heated. Disruption of bonding within the peptide chain may result in a less thermally stable tertiary structure without allowing for significant release of peptide fragments, while randomization of the crosslinking between fibrils (though breakage and reformation) may create a structure that is more prone to uncoiling through heating. The reduction in denatured collagen content during long term storage of the high dose gamma tendons (30G, 55G) suggests that some of the bonds broken during irradiation are reformed over time. Due to the relatively low levels of denatured collagen present in all conditions, these changes may only be apparent at the higher doses.

In contrast, the changes in mechanical properties following irradiation were not dose dependant over the dose ranges tested. The gross material properties of the tendon tissue are largely dependent on the hierarchical structure with large numbers of individual fibres bundled together to increase strength.^37^ While damage to the peptide chains might cause some reduction in the strength of individual fibres, many covalent linkages between fibre bundles would still be present and may have increased in number due to the presence of water during irradiation. The formation of new crosslinks may have masked any irradiation induced dose‐dependent increase in chain scission by concurrently reinforcing fibrils as the dosage increased.

It is important to note that, although irradiation affected the mechanical properties of the d‐pSFT, the grafts still had mechanical properties similar to the native human ACL. Although the study presented here uses a strain rate lower than many studies investigating the mechanical properties of ACLs, an increased strain rate would most likely only serve to increase parameters such as Young's modulus. Hence, values reported stand well in comparison to those of the native ACL. A study by Chandrashekar et al. reported average values for the UTS (24.36 ± 9.38 MPa), failure strain (0.28 ± 0.07), and Young's modulus (113 ± 45).^38^ Elsewhere, UTS has been reported to be higher (37.8 ± 9.3 MPa^39^). In this study, it was shown that the d‐pSFT subject to 30 kGy gamma irradiation and stored for 12 months had values of UTS (39.96 ± 3.68 MPa), failure strain (0.29 ± 0.03), and Young's modulus (243 ± 12 MPa). This suggests that d‐pSFT terminally sterilized with 30 kGy gamma irradiation would provide ample mechanical support in place of the native ACL.

## CONCLUSION

Several different irradiation conditions were tested with a previously developed decellularized porcine superflexor tendon graft to determine their suitability as terminal sterilization methods with respect to biological and biomechanical properties. Biomechanical properties were altered for all irradiation conditions compared PAA alone, but there was no dose dependency. Denatured collagen content and thermal properties of the grafts showed dose dependent responses to irradiation, suggesting changes to the tertiary protein structure at higher doses which did not affect mechanical integrity. The d‐pSFT grafts were found to be biocompatible in *in vitro* contact tests for all irradiation treatments and time periods tested. Low doses of both gamma and E‐beam irradiation showed no significant benefits compared to the standard minimum 25 kGy Gamma dose (30G). We suggest that irradiation with a minimum of 25 kGy gamma may be the most appropriate dose for terminal sterilization of these decellularized pSFT grafts. Storage of the grafts for 12 months at 4°C poststerilization was also found to have no negative effects on the grafts, demonstrating their suitability as an ‘off‐the‐shelf’ solution for ACL repair.
